# Randomised phase II trial of irinotecan plus cisplatin *vs* irinotecan, cisplatin plus etoposide repeated every 3 weeks in patients with extensive-disease small-cell lung cancer

**DOI:** 10.1038/sj.bjc.6604233

**Published:** 2008-02-05

**Authors:** I Sekine, H Nokihara, K Takeda, Y Nishiwaki, K Nakagawa, H Isobe, K Mori, K Matsui, N Saijo, T Tamura

**Affiliations:** 1 Division of Internal Medicine and Thoracic Oncology, National Cancer Center Hospital, Tokyo, Japan; 2 Department of Clinical Oncology, Osaka City General Hospital, Osaka, Japan; 3 Division of Thoracic Oncology, National Cancer Center Hospital East, Kashiwa, Japan; 4 Department of Medical Oncology, Kinki University School of Medicine, Sayama, Japan; 5 Department of Pulmonary Disease, National Hospital Organization Hokkaido Cancer Center, Sapporo, Japan; 6 Department of Thoracic Diseases, Tochigi Prefectural Cancer Center, Utsunomiya, Japan; 7 Department of Internal Medicine, Osaka Prefectural Medical Center for Respiratory and Allergic Diseases, Habikino, Japan

**Keywords:** small-cell lung cancer, chemotherapy, irinotecan, etoposide, three drug combination

## Abstract

Patients with previously untreated extensive-disease small-cell lung cancer were treated with irinotecan 60 mg m^−2^ on days 1 and 8 and cisplatin 60 mg m^−2^ on day 1 with (*n*=55) or without (*n*=54) etoposide 50 mg m^−2^ on days 1–3 with granulocyte colony-stimulating factor support repeated every 3 weeks for four cycles. The triplet regimen was too toxic to be considered for further studies.

Small-cell lung cancer (SCLC), which accounts for approximately 14% of all malignant pulmonary tumours, is an aggressive malignancy with a propensity for rapid growth and early widespread metastases (Jackman and Johnson, 2005). A combination of cisplatin and etoposide (PE) has been the standard treatment, with response rates ranging from 60 to 90% and median survival times (MSTs) from 8 to 11 months in patients with extensive disease (ED)-SCLC (Fukuoka *et al*, 1991; Roth *et al*, 1992). A combination of irinotecan and cisplatin (IP) showed a significant survival benefit over the PE regimen (MST: 12.8 *vs* 9.4 months, *P*=0.002) in a Japanese phase III trial for ED-SCLC (Noda *et al*, 2002), although another phase III trial comparing these regimens failed to show such a benefit (Hanna *et al*, 2006). Thus, irinotecan, cisplatin and etoposide are the current key agents in the treatment of SCLC. A phase II trial of the three agents, IPE combination, in patients with ED-SCLC showed a promising antitumour activity with a response rate of 77%, complete response (CR) rate of 17% and MST of 12.9 months (Sekine *et al*, 2003).

We have developed these IP and IPE regimens in a 4-week schedule where irinotecan was given on days 1, 8 and 15. The dose of irinotecan on day 15, however, was frequently omitted because of toxicity in both regimens (Noda *et al*, 2002; Sekine *et al*, 2003). The objectives of this study were to evaluate the toxicities and antitumour effects of IP and IPE regimens in the 3-week schedule in patients with ED-SCLC and to select the right arm for subsequent phase III trials.

## PATIENTS AND METHODS

### Patient selection

Patients were enrolled in this study if they met the following criteria: (1) a histological or cytological diagnosis of SCLC; (2) no prior treatment; (3) measurable disease; (4) ED, defined as having distant metastasis or contralateral hilar lymph node metastasis; (5) performance status of 0–2 on the Eastern Cooperative Oncology Group (ECOG) scale; (6) predicted life expectancy of 3 months or longer; (7) age between 20 and 70 years; (8) adequate organ function as documented by a white blood cell (WBC) count ⩾4.0 × 10^3^ *μ*l^−1^, neutrophil count ⩾2.0 × 10^3^ *μ*l^−1^, haemoglobin ⩾9.5 g dl^−1^, platelet count ⩾100 × 10^3^ *μ*l^−1^, total serum bilirubin⩽1.5 mg dl^−1^, hepatic transaminases⩽100 IU l^−1^, serum creatinine ⩽1.2 mg dl^−1^, creatinine clearance ⩾60 ml min^−1^, and PaO_2_ ⩾60 torr; and (9) providing written informed consent.

Patients were not eligible for the study if they had any of the following: (1) uncontrollable pleural, pericardial effusion or ascites; (2) symptomatic brain metastasis; (3) active infection; (4) contraindications for the use of irinotecan, including diarrhoea, ileus, interstitial pneumonitis and lung fibrosis; (5) synchronous active malignancies; (6) serious concomitant medical illness, including severe heart disease, uncontrollable diabetes mellitus or hypertension; or (7) pregnancy or breast feeding.

### Treatment schedule

In the IP arm, cisplatin, 60 mg m^−2^, was administered intravenously over 60 min on day 1 and irinotecan, 60 mg m^−2^, was administered intravenously over 90 min on days 1 and 8. Prophylactic granulocyte colony-stimulating factor (G-CSF) was not administered in this arm. In the IPE arm, cisplatin and irinotecan were administered at the same dose and schedule as the IP arm. In addition, etoposide, 50 mg m^−2^, was administered intravenously over 60 min on days 1–3. Filgrastim 50 *μ*g m^−2^ or lenograstim 2 *μ*g kg^−1^ was subcutaneously injected prophylactically from day 5 to the day when the WBC count exceeded 10.0 × 10^3^ *μ*l^−1^. Hydration (2500 ml) and a 5HT_3_ antagonist were given on day 1, followed by an additional infusion if indicated in both arms. These treatments were repeated every 3 weeks for a total of four cycles.

### Toxicity assessment, treatment modification and response evaluation

Toxicity was graded according to the NCI Common Toxicity Criteria version 2.0.

Doses of anticancer agents in the following cycles were modified according to toxicity in the same manner in both arms. Objective tumour response was evaluated according to the Response Evaluation Criteria in Solid Tumors (RECIST) (Therasse *et al*, 2000).

### Study design, data management and statistical considerations

This study was designed as a multi-institutional, prospective randomised phase II trial. This study was registered on 6 September 2005 in the University hospital Medical Information Network (UMIN) Clinical Trials Registry in Japan (http://www.umin.ac.jp/ctr/index.htm), which is acceptable to the International Committee of Medical Journal Editors (ICMJE) (http://www.icmje.org/faq.pdf). The protocol and consent form were approved by the Institutional Review Board of each institution. Patient registration and randomisation were conducted at the Registration Center. No stratification for randomisation was performed in this study. The sample size was calculated according to the selection design for pilot studies based on survival (Liu *et al*, 1993). Assuming that (1) the survival curve was exponential for survivals; (2) the MST of the worse arm was 12 months and that of the better arm was 12 months × 1.4; (3) the correct selection probability was 90%; and (4) additional follow-up in years after the end of accrual was 1 year, the estimated required number of patients was 51 for each arm. Accordingly, 55 patients for each arm and their accrual period of 24 months were planned for this study.

The dose intensity of each drug was calculated for each patient using the following formula as previously described: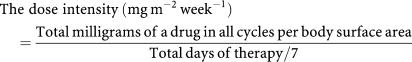 where total days of therapy is the number of days from day 1 of cycle 1 to day 1 of the last cycle plus 21 days for both arms (Hryniuk and Goodyear, 1990).

Differences in the reason for termination of the treatment and the frequencies of grade 3–4 toxicities were assessed by χ tests. Survival was measured as the date of randomisation to the date of death from any cause or the date of the most recent follow-up for overall survival and to the date of disease progression or the date of death for progression-free survival (PFS). The survival of the arms was estimated by the Kaplan–Meier method and compared in an exploratory manner with log-rank tests (Armitage *et al*, 2002).

## RESULTS

### Patient characteristics

From March 2003 to May 2005, 55 patients were randomised to IP and 55 patients to IPE. One patient in the IP arm was excluded because the patient was ineligible and did not receive the study treatment. The remaining 109 patients were included in the analyses of toxicity, tumour response and patient survival. There were no differences between the two arms in any demographic characteristics listed (Table 1).

### Treatment delivery

Treatment was well tolerated with respect to the number of cycles delivered in both arms (Table 2). Among reasons for termination of the treatment, disease progression was noted in nine (17%) patients in the IP arm and in two (4%) patients in the IPE arm, whereas toxicity was noted in three (6%) patients in the IP arm and 13 (24%) patients in the IPE arm (*P*=0.013) (Table 2). The dose of irinotecan on day 8 was omitted in 35 (18%) cycles in the IP arm and 37 (17%) cycles in the IPE arm (Table 2). The total dose and dose intensity of cisplatin and etoposide were similar between the IP and IPE arms in the present study (Table 3).

### Toxicity

The myelotoxicity was more severe in the IPE arm (Table 4). Grade 3 febrile neutropaenia was noted in 5 (9%) patients in the IP arm and 17 (31%) patients in the IPE arm (*P*=0.005). Packed red blood cells were transfused in 4 (7%) patients in the IP regimen and 14 (26%) patients in the IPE regimen (*P*=0.011). Platelet concentrates were needed in none in the IP regimen and 2 (4%) patients in the IPE regimen (*P*=0.16). Grade 3–4 diarrhoea was observed in 8 (15%) patients in the IP arm and 13 (24%) patients in the IPE arm (*P*=0.262). Grade 3–4 fatigue was more common in the IPE arm with marginal significance (2 *vs* 11%, *P*=0.054). The severity of other non-haematological toxicities did not differ significantly between the arms. No treatment-related death was observed in this study.

### Response, treatment after recurrence and survival

**Figure 1:**
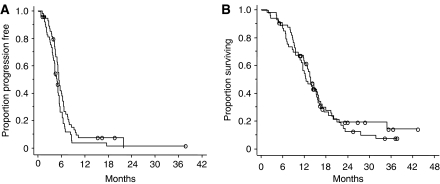
Progression-free survival (**A**) and overall survival (**B**). Thick line indicates the IPE regimen and thin line indicates the IP regimen.

Four CRs and 37 partial responses (PRs) were obtained in the IP arm, resulting in the overall response rate of 76 with 95% confidence interval (CI) of 65–87%, whereas six CRs and 42 PRs were obtained in the IPE arm, and the overall response rate was 87% with a 95% CI of 79–96% (*P*=0.126). Median PFS was 4.8 months (95% CI, 4.0–5.6) in the IP and 5.4 months (95% CI, 4.8–6.0) in the IPE arm (*P*=0.049) (Figure 1A). After recurrence, 22 (44%) patients in the IP arm and 8 (16%) patients in the IPE arm received etoposide-containing chemotherapy. The MST and 1-year survival rate were 12.4 months (95% CI, 9.7–15.1) and 54.8% (95% CI, 41.4–68.2%) in the IP and 13.7 months (95% CI, 11.9–15.5) and 61.5% (95% CI, 48.6–74.4%) in the IPE arm (*P*=0.52), respectively (Figure 1B).

## DISCUSSION

This study showed that the IPE regimen in a 3-week schedule with CSF support produced a promising response rate, PFS and overall survival. Haematological toxicity in the IPE arm, however, was very severe in spite of the G-CSF support with the grade 3 febrile neutropaenia noted in 31% of patients.

In comparison between the 3-week IPE regimen in this study and the 4-week IPE regimen in the previous study, the delivery of cisplatin and etoposide was improved in the 3-week IPE regimen when compared with the 4-week IPE regimen at the cost of the irinotecan total dose. The response rate and MST were 87% and 13.7 months, respectively, in the 3-week IPE regimen and 77% and 12.9 months in the previous 4-week schedule, and toxicity profiles were comparable to each other (Sekine *et al*, 2003).

The MST of 12.4 months in the IP arm in this study was comparable to that of the previous phase III study, with an MST of 12.8 months (Noda *et al*, 2002). Thus, this study showed the reproducible excellent survival outcome of patients with ED-SCLC who were treated with the IP combination. In contrast, a recent American phase III study of the PE regimen *vs* IP regimen failed to show the superiority of the IP regimen to the PE regimen; the MST for the PE regimen was 10.2 months and that for the IP regimen was 9.3 months (Hanna *et al*, 2006). The discrepancy between the Japanese and American trials may be explained by the different cisplatin dose schedules; cisplatin was delivered at a dose of 60 mg m^−2^ on day 1 every 3 or 4 weeks in the Japanese trials, whereas cisplatin was delivered at a dose of 30 mg m^−2^ on days 1 and 8 every 3 weeks in the American one. A platinum agent administered at divided doses was associated with poor survival in patients with ED-SCLC in our previous randomised phase II study (Sekine *et al*, 2003).

The issue of adding further agents to the standard doublet regimen has been investigated in patients with ED-SCLC. The addition of ifosfamide or cyclophosphamide and epirubicin to the cisplatin and etoposide combination produced a slight survival benefit, but at the expense of greater toxicity (Loehrer *et al*, 1995; Pujol *et al*, 2001). Phase III trials of cisplatin and etoposide with or without paclitaxel showed unacceptable toxicity with 6–13% toxic deaths in the paclitaxel-containing arm (Mavroudis *et al*, 2001; Niell *et al*, 2005). The results in these studies and the current study are consistent in the increased toxicity despite the G-CSF support and no definite survival benefit in the three or four drug combinations over the standard doublet in patients with ED-SCLC.

In conclusion, the IPE regimen was marginally more effective than the IP regimen, but was too toxic despite the administration of prophylactic G-CSF.

## Figures and Tables

**Table 1:** Patient characteristics

	**IP (*n*=54)**	**IPE (*n*=55)**
*Sex*		
Female	11	8
Male	43	47
		
*Age (years)*		
Median (range)	63 (42–70)	62 (48–70)
		
		
*PS*		
0	11	12
1	42	41
2	1	2
		
*Weight loss*		
0–4%	38	43
5–9%	10	10
⩾10%	6	2

**Table 2:** Treatment delivery

	**IP (*n*=54)**	**IPE (*n*=55)**
	**No. (%)**	**No. (%)**
*Number of cycles delivered*		
6^a^	—	1 (2)
4	41 (76)	36 (65)
3	6 (11)	6 (11)
2	3 (6)	6 (11)
1	4 (7)	6 (11)
		
*Reasons for termination of the treatment* ^†^		
Completion	40 (74)	35 (64)
Disease progression	9 (17)	2 (4)
Toxicity	3 (6)	13 (24)
Patient refusal	2 (4)	4 (7)
Others	0 (0)	1 (2)
		
Total number of cycles delivered	192 (100)	186 (100)
Total number of omission on day 8	35 (18)	37 (17)
Total number of cycles with dose reduction	28 (15)	31 (17)

^†^*P*=0.013 by *χ*^2^ test.

a Protocol violation.

**Table 3:** Total dose and dose intensity

	**3-week regimens in this study**		**4-week regimen^a^**
	**IP (*n*=54)**	**IPE (*n*=55)**	**IPE (*n*=30)**
	**Median (range)**	**Median (range)**	**Median (range)**
*Total dose (mg m^−2^)*			
Cisplatin	240 (60–240)	240 (60–360)	240 (60–240)
Irinotecan	420 (60–480)	390 (60–720)	563 (60–720)
Etoposide	0	600 (150–900)	600 (150–600)
			
*Dose intensity (mg m^−2^ week^−1^)*			
Cisplatin	19 (14–25)	20 (16–34)	15 (12–15)
Irinotecan	33 (14–40)	35 (15–55)	35 (19–45)
Etoposide	0	48 (34–68)	37 (28–38)

a From our previous study (Sekine *et al*, 2003).

**Table 4:** Grade 3–4 toxicities

	**IP (*n*=54)**			**IPE (*n*=55)**		
	**Grade 3**	**4**	**3+4 (%**)	**Grade 3**	**4**	**3+4 (%)**
Leukocytopaenia	9	1	10 (19)	18	11	29 (53)^*^
Neutropaenia	17	11	28 (52)	24	28	52 (95)^*^
Anaemia	18	0	18 (25)	16	9	25 (45)
Thrombocytopaenia	2	0	2 (4)	13	0	13 (13)^†^
Febrile neutropaenia	5	0	5 (9)	17	0	7 (13)
Diarrhoea	8	0	8 (15)	11	2	13 (24)
Vomiting	4	0	4 (7)	3	0	3 (5)
Fatigue	1	0	1 (2)	5	1	6 (11)^‡^
Hyponatraemia	9	3	12 (22)	11	2	13 (24)
AST elevation	0	0	0 (0)	3	0	3 (5)
CRN elevation	1	0	1 (2)	0	0	0 (0)

^*^P<0.001; ^†^*P*<0.01; and ^‡^*P*=0.054 by *χ*^2^ test.
